# Perspective: science and the future of livestock industries

**DOI:** 10.3389/fvets.2024.1359247

**Published:** 2024-01-11

**Authors:** Graeme B. Martin

**Affiliations:** The UWA Institute of Agriculture and UWA School of Agriculture and Environment, The University of Western Australia, Crawley, WA, Australia

**Keywords:** animal-sourced food, methane emissions, global heating, reproduction, animal welfare, stress, nutrition, health

## Abstract

Since the 1990s, livestock industries have been forced to respond to major pressures from society, particularly with respect to methane emissions and animal welfare. These challenges are exacerbated by the inevitability of global heating and the effects it will have on livestock productivity. The same challenges also led to questions about the value of animal-sourced foods for feeding the world. The industries and the research communities supporting them are meeting those challenges. For example, we can now envisage solutions to the ruminant methane problem and those solutions will also improve the efficiency of meat and milk production. Animal welfare is a complex mix of health, nutrition and management. With respect to health, the ‘One Health’ concept is offering better perspectives, and major diseases, such as helminth infection, compounded by resistance against medication, are being resolved through genetic selection. With respect to nutrition and stress, ‘fetal programming’ and the epigenetic mechanisms involved offer novel possibilities for improving productivity. Stress needs to be minimized, including stress caused by extreme weather events, and solutions are emerging through technology that reveals when animals are stressed, and through an understanding of the genes that control susceptibility to stress. Indeed, discoveries in the molecular biology of physiological processes will greatly accelerate genetic progress by contributing to genomic solutions. Overall, the global context is clear – animal-sourced food is an important contributor to the future of humanity, but the responses of livestock industries must involve local actions that are relevant to geographical and socio-economic constraints.

## Introduction

In keeping with the theme of this collection, I offer my perspective on the major challenges that are confronting livestock industries. This paper is based on keynote papers that I was invited to present at two meetings held at the Shaheed Benazir Bhutto University of Veterinary and Animal Sciences (SBBUVAS; Sakrand, Sind, Pakistan): (i) *First International Symposium on Animal Welfare and One Health* (May 2022); and (ii) *Animal Production and Food Security – Identifying Challenges and Finding a Way Forward* (April 2023).

Some forecasting is needed but, as we look further into the future, forecasting becomes increasingly risky because of unpredictable changes in technology, not to mention geopolitical upheaval. I will therefore limit myself to the next three decades for which we can be confident about three issues: (1) we will need to feed about 50% more people with shrinking resources; (2) global heating will affect livestock production systems; (3) societies and therefore markets will continue to pressure livestock industries to be ‘clean, green and ethical’ (CGE). Finally, I will suggest opportunities for research.

### Feeding another 3 billion people by 2050

Human population growth became a focus of concern with the so-called ‘population bomb’ paper in which the number of people on the planet was predicted to reach infinity on Friday, 13 November, 2026 (1). As I used to tell my students, even half of infinity was not possible! Happily, the outlook has become far less dramatic because eminent demographers, such as Sarah Harper, have shown that a worldwide decline in Total Fertility Rate (TFR) will limit the maximum population to about 12 billion (2). This news might be good, but we are still on track for 11 billion people by 2050, having passed 8 billion in late 2023.

It is therefore inevitable that, within 30 years, we will need to feed an extra 3 billion people. This task is made significantly more difficult because the resource base for food production is shrinking as the amount of arable land per person diminishes due to population growth, city expansion over farmland, and land degradation (3). Moreover, global heating is already reducing food security and current predictions suggest this problem will become worse (4, 5).

### Global heating will affect livestock productivity

Livestock enterprises are often seen as more resistant to global heating because the homeostatic physiology of animals can easily cope with, and even adapt to, an *average* temperature increase of say 2°C, especially if they are aided by natural selection, controlled breeding programs or environmental management. However, the real danger of global heating is probably changes in precipitation patterns (droughts, floods) with major effects on the availability of drinking water and feedstuffs (6, 7). More recent situation analyses have re-enforced the indirect effects (reduced productivity of pastures, forage crops and feeds) and also outlined direct effects on growth, welfare, reproduction and animal health (8, 9). Animal health is often ignored, yet it is clear that shifts in climate zones will affect the persistence and abundance of disease vectors and parasites, leading to increases in disease severity (10–12).

Global heating will also increase the frequency and magnitude of extreme weather events such as heat waves. Heat waves have long been considered a fertility risk in grazing livestock, particularly in male sheep and goats because a stress event in summer will have deleterious effects on sperm produced one spermatogenic cycle later, during the normal autumn breeding season. We now know that an increase in testis temperature reduces blood flow, thus restricting the supply of nutrients, regulatory hormones, and oxygen (13). Female reproduction is also disrupted by heat stress, as is animal welfare, such that, in Australia, shade is acknowledged by industry as the next frontier in the management of grazing animals [review: (14)]. It is this no surprise that Björkbom (15) argues logically that animal welfare must be included in policies targeting food sustainability.

### Societal and market pressures are affecting livestock management

Changes in society and thus the marketplace led to the development of a vision for ‘clean, green and ethical’ (CGE) livestock management -: ‘clean’ involves adoption of practices that minimize the treatment of animals with hormones, drugs and chemicals; ‘green’ involves ensuring that the industry is environmentally sustainable; ‘ethical’, involves avoiding practices that compromise animal welfare. Importantly, these three principles are not independent – for example, ‘ethical’ considerations are also relevant to the ‘clean’ and ‘green’ aspects of management. Equally importantly, the CGE principles apply to all participants in the supply chain, from producers to transporters to processors.

In the beginning, in 2002, the CGE concept focussed specifically on sheep reproduction and it was placed before thousands of sheep producers in Australia. In 2004, it was presented to an international science audience in Brazil. It has since been discussed at dozens of international meetings and workshops in many countries, and now seems to be accepted world-wide.

Recently, 20 years of discoveries in reproductive biology were accommodated in an update (16). In brief: the foundation of CGE management is understanding how the reproductive system responds to environmental factors, so those factors can be manipulated to improve reproductive outcomes. The primary factors are photoperiod, nutrition, and pheromones, to which we now need to add stressors, including extreme weather events, as discussed above. In females, we now know that metabolic signals, including the adipokines, act directly on ovarian follicles to affect the balance between cell proliferation and apoptosis (atresia) that, in turn, determines ovulation rate. In males, the responses to metabolic signals involve processes in the brain that control gonadotrophin secretion (the kisspeptin system) and processes in the testis (eg, non-coding RNAs) that affect the balance between proliferation and apoptosis in germ cells. This proliferation-apoptosis balance can also be affected during prenatal development, when undernutrition or stress seem to elicit epigenetic changes in developing gonads that affect offspring fertility in adult life. Indeed, the whole field of ‘fetal programming’, or developmental origins of health and disease (DoHaD) has exploded since the first CGE paper was published in 2004, with evidence gathering for a lengthening list of productivity measures that are affected by epigenetic effects on sperm, oocytes, embryos and fetuses [eg., (17–20)]. For postnatal life, it has become clear that puberty can be advanced by accelerating the accumulation of muscle as well as fat, a major advantage for meat production systems. With respect to pheromones (‘male effect’), we now better understand the brain responses (the kisspeptin system again) but, most importantly, we have learned that the response of ewes to the ram signal involves cell division in memory centers, and thus ‘olfactory memory’ [review: (16)].

Over the last two decades, the CGE concept has been applied beyond sheep to include other livestock systems, including industries based on monogastric species.

## What is the future of food produced from livestock?

As CGE management was being developed and promoted, livestock industries worldwide were being subjected to a broader examination, beginning with the publication by FAO of *Livestock’s Long Shadow* in which the overall conclusion was that livestock industries are not sustainable (21). Heated debate followed. In 2014, Eisler et al. (22) re-addressed many of the issues and presented a more balanced perspective that arose as the consensus from an international workshop run under the auspices of the *Worldwide Universities Network*. The authors proposed that ‘ruminant livestock could help to feed the world without destroying the planet’, but also acknowledged several major issues that needed attention. Then, in 2022, the unnecessarily controversial issue of the value of meat and milk as human food, as well as the environmental impacts of livestock production systems, were addressed in the *Dublin Declaration of Scientists on the Societal Role of Livestock* (23), with editorial support provided by Ederer and Leroy (24). *Note:* in the interests of transparency, I did not participate in the Dublin meeting, but I did subsequently sign the declaration.

Some of the issues listed by Eisler and colleagues (22) are common to the arguments raised in *The Dublin Declaration*, and several fall under the umbrella of CGE livestock management. The exceptions are those that are related to broader human food systems. Here, I will attempt to integrate the major aspects of these three sets of complimentary perspectives.

### ‘Clean-ethical’ – animal health, nutrition and welfare are essential for production efficiency

Clearly, health is at the heart of ‘ethical’ animal management. In recent times, we have seen the rise to prominence of ‘One Health’, a concept that can be traced back to 1964, if not earlier, when the veterinarian Calvin Schwabe, used the term “One Medicine” in a veterinary medical textbook. It is no surprising that the concept was given prominence in ‘Steps to Sustainable Livestock’ (22).

A focus on health is ethically essential but, over the decades, we have become too reliant on medical solutions, leading to excessive usage of, for example, antibiotics and anthelmintics. Clearly, ‘ethical’ intersects with ‘clean’ because of the risk of food residues, but a more acute problem is the development of resistance by pathogens. Antibiotic resistance is often in the headlines but, worldwide, we have also witnessed the evolution of resistance to anthelmintic medication (25) documented most recently in Sweden (26).

Until the arrival of anthelmintics, production systems relied on natural resistance (survival of the fittest) plus management of infection by rotational grazing to break the helminth life cycle. In effect, anthelmintics allowed susceptible animals to avoid being culled and to breed. Mismanagement of anthelmintics exacerbated the problem (25). Breeding for resistance to infection directly reverses this process, improving the health, welfare and productivity of animals, while reducing our reliance on medication, thus helping the industry to become ‘cleaner’ (27).

A critical aspect of animal welfare is avoiding stress. One seemingly inevitable stressor is extreme weather events. Livestock managers might not be able to control the weather, but they can provide shade and shelter to reduce the impact of cold and heat (14). Moreover, genetic solutions are feasible because we are beginning to understand the genes that determine and animal’s response to a stressor and can therefore breed animals that are less reactive (28). Another major impediment is that, except in extreme situations, livestock managers cannot know when their animals are uncomfortable. Technological solutions are on the horizon, such as the subcutaneous sensor that can detect temperature rhythms that respond to stress events (29).

### ‘Green’ – environmental footprint

Methane emitted by ruminants was among the problems highlighted in *Livestock’s Long Shadow*. At that time, our thinking was constrained by three pre-conceptions: (a) methane production in the rumen was essential for taking up hydrogen ions and preventing acidosis, so blocking the process would kill the animal; (b) methane production was not a heritable trait; (c) feed additives could not reduce methane synthesis. The period 2006–2014 saw major advances in methane science, and all three pre-conceptions were rejected – we now have estimates of heritability (30, 31) and a variety of novel forages and dietary additives that can reduce emissions [review: (32)]. Moreover, blocking methane synthesis is not detrimental for the animal (33) – in fact, it improves animal efficiency because carbon that would have escaped by eructation is redirected into production (34). In other words, reducing methane production is a ‘win-win’ situation. Finally, researchers developed the critical concept of ‘methane efficiency’ thus providing an industry driver for reducing the mass of methane produced per unit mass of product. For example, methane efficiency is improved by improving health (11, 35).

Meanwhile, the Global Warming Potential (GWP) of methane was being re-assessed by factoring in the rate of methane emission over a period of time and the rate of degradation of emitted methane. The outcome has been an argument for replacing the 100-year Global Warming Potential (GWP100) with GWP* (36) as a measure of the actual warming potential of methane instead of relying on its CO_2_ equivalence [review: (37)].

The ruminant methane problem is therefore largely resolved (38). Moreover, any emissions that persist will be trivial compared to the methane in ‘fugitive emissions’ – an obfuscation for the greenhouse gasses (GHG) that escape during extraction of coal and gas – let alone the total emissions from the fossil-fuel energy sector. In this context, it is worth repeating some of the text in the *Emirates Declaration on Sustainable Agriculture, Resilient Food Systems, and Climate Action*^1^:

- “Recognizing that unprecedented adverse climate impacts are increasingly threatening the resilience of agriculture and food systems …”;
- “Noting that agriculture and food systems are fundamental to the lives and livelihoods of billions of people, including smallholders, family farmers … and food workers ….”
- The clarity and importance of these statements resonates with those of us working in the agriculture/food sector across many countries, as do the following statements:
- “We affirm that agriculture and food systems must urgently adapt and transform in order to respond to the imperatives of climate change”;
- “Maximize the climate and environmental benefits … associated with agriculture and food systems by … shifting from higher greenhouse gas-emitting practices to more sustainable production and consumption approaches ….”

It is notable that methane emissions from ruminant livestock are not mentioned specifically. Considering the location of COP28 and its management structure, and that the full COP28 Declaration was the first time in 28 COP meetings that the words “fossil fuel” have been included, there seems to be a better balance. This outcome seems like justice because small farmers are an easier target than the massive fossil fuel companies that sent two thousand lobbyists to COP28. The improved balance in the Declaration is a success for the excellent research that has been done on the various aspects of ruminant methane over the past two decades.

### ‘Ethical’ – genotypes should be chosen that are adapted to local challenges

In the quest for a quantum leap in productivity, exotic genotypes are often seen as a simple solution. The folly of this approach, and the ethical issues raised, are particularly evident when Holstein dairy cattle are transferred from temperate into tropical regions, even when it is well known that the animals will have poor resistance to ambient heat, local diseases and parasites, and be poorly adapted to local forages. In the animals that survive, production is much lower than expected while costs are significantly increased for medication, housing and feed. The farmers that receive these animals also become highly stressed.^2^

The solution is return the focus to indigenous genotypes that can already cope with local conditions and improve productivity by carefully planned use of reproductive technology and genetic and genomic tools (39, 40). The simplicity of this approach is demonstrated by the introduction of Holstein genes to improve milk production in Sanga cattle in Ghana (41).

Strategies based on indigenous genotypes will also reduce the loss of diversity in genetic resources that will probably be needed for adaptation to challenges such as the above-mentioned threats from global heating to growth, productivity, welfare, reproduction and health (42).

## Issues related to human nutrition

Two of the ‘steps toward sustainable livestock’ proposed by Eisler and colleagues (22) were for more human-edible grain to be directed away from livestock systems, and for a global re-assessment of the human diet with a view to improving human health, partly by reducing meat consumption in some societies.

### Less human food should be consumed by livestock

Early estimates from FAO suggested that a third of human-edible grain is fed to livestock rather than humans (22), but a more recent study suggests that the proportion is considerably smaller (43). This issue was addressed in *The Dublin Declaration* (23, 24) and subsequently in more detail, in the Australian context, by Pethick and colleagues (44), who argued for a balanced perspective. Indeed, it is neither practical nor efficient to confine ruminants to areas where crops cannot be grown – many highly successful production systems for human food involve livestock-crop rotations. Similarly, grain supplements can significantly improve production efficiency and the utilization of food waste and low-value forages.

These nuances aside, we do need to maintain pressure on ruminant production systems to minimize the consumption of human-edible grain in Total Mixed Rations. The inefficiency is obvious – the evolution of ruminants enables them to digest forages, thus converting a resource of no nutritional value for humans to meat and milk that are of exceptional nutritional value. After all, this ability was a driver of their domestication. Moreover, forage-based ruminant systems are best for minimizing the amount of GHG produced per kg of human-edible food (45).

### Healthy diets for humans, with a smaller meat component

The ‘CGE’ concept, especially the detrimental effects of livestock industries on the environment (*Livestock’s Long Shadow*), sparked fervour in the vegetarian/vegan food movement leading to somewhat extreme proposals such as completely abandoning animals as a source of human food. Eisler et al. (22) reminded us that we cannot ignore the cultural value of livestock, and also defended the value of animal protein in the human diet. This latter point was addressed in detail in 2022 in *The Dublin Declaration* (23, 24), where evidence was presented showing clearly that meat is a nutrient-dense source of high-quality protein and micronutrients that can be safely consumed by humans. Indeed, the detrimental effects of stunting on development in children is well documented, as is the role of meat in avoiding such problems (eg, 46, 47).

That said, there is a global issue in balance and equity when perhaps a billion people are undernourished and perhaps a billion people are obese. This imbalance is stark when about 12% of people in the US seem to account for half of all beef consumption in that country (48).

## Opportunities for research in CGE management

The livestock industries are dynamic and, while they have a robust future in feeding and clothing the world, they will have to evolve in response to changes in the societal, economic and physical environment in which they operate. The demand for animal protein is expanding but the planet is not. Responses to these challenges will always be founded on solid science, and the solutions will be diverse and multidisciplinary, as will the opportunities for research. Rather than offer an impossible list, I will confine my suggestions to research in reproduction: (i) Olfactory memory in the context of both the male effect and mother-young bonding; (ii) DoHaD and epigenetics, perhaps the ‘hottest’ current topic in reproductive biology; (iii) Embryo mortality, traditionally a very difficult research topic but now vulnerable to new tools for quantifying and investigating the problem; (iv) Postnatal survival, often dismissed as a problem confined to multiple births, but multiple births will be essential in future production systems, so it is time to take on the challenge (49). Importantly, the 100% CGE model (16) is not going to be applicable to all industries in all geographical or socio-economic environments, but individual aspects of the model can be introduced in a planned process (50, 51) that needs to be supported by local applied research.

## Conclusion

The global context is clear – livestock science must respond to increasing demand for animal-based food in the face of limited resources and global warming. The need for local action is also clear because the solution to any problem must have local context, fitting the socio-economic environment, cultural mores, and physical geography. A wide variety of solutions is needed to make livestock industries more ‘clean, green and ethical’, as well as more productive. Many of these solutions will come from big data, biological technologies and genomic breeding, and present many exciting, relevant opportunities for research students.

## Author contributions

The author confirms being the sole contributor of this work and has approved it for publication.

## Footnotes

^1^ https://www.cop28.com/en/food-and-agriculture

^2^ https://www.rspca.org.au/media-centre/news/2019/rspca-calls-urgent-action-address-major-live-export-loophole
